# A Low-Cost, Low-Power Water Velocity Sensor Utilizing Acoustic Doppler Measurement

**DOI:** 10.3390/s22197451

**Published:** 2022-09-30

**Authors:** Stephen Catsamas, Baiqian Shi, Boris Deletic, Miao Wang, David T. McCarthy

**Affiliations:** BoSL Water Monitoring and Control, Department of Civil Engineering, Monash University, Melbourne, VIC 3800, Australia

**Keywords:** acoustic Doppler velocimetry, field verification, low cost, low power, open hardware, real-time environmental monitoring, sensor design, stormwater monitoring, urban water, water velocity

## Abstract

Current commercial sensors to monitor water flow velocities are expensive, bulky, and require significant effort to install. Low-cost sensors open the possibility of monitoring storm and waste water systems at a much greater spatial and temporal resolution without prohibitive costs and resource investment. To aid in this, this work developed a low-cost, low-power velocity sensor based on acoustic Doppler velocimetry. The sensor, costing less than 50 USD is open-source, open-hardware, compact, and easily interfaceable to a wide range of data-logging systems. A freely available sensor design at this price point does not currently exist, and its novelty is in enabling high-resolution real-time monitoring schemes. The design is capable of measuring water velocities up to 1200 mm/s. The sensor is characterised and then verified in an in-field long-term test. Finally, the data from this test are then used to evaluate the performance of the sensor in a real-world scenario. The analysis concludes that the sensor is capable of effectively measuring water velocity.

## 1. Introduction

The velocity of stormwater, wastewater, and surface water is an essential parameter that requires continuous monitoring to estimate the discharge volume and conduct further analyses, such as pollution load estimation [1], flow and water quality modelling [2,3], and normalisation of pathogen concentrations in wastewater [4,5]. Current sensors available on the market to measure water velocity in storm and waste water systems are expensive, are high-power, are not available freely as open-source or open-hardware, and/or require significant preparation and overhead to setup and maintain [6,7,8]. Such sensors are not feasible for research and water operations where the objective is to collect higher spatial resolution data to, for example, further improve the accuracy of water models [9] and the performance of sewer surveillance programs [10]. Other drivers for low-cost velocity sensors include the improvement in existing flow measurement stations that simply rely on stage-discharge relationships, where the determination and updating of flow rating curves is costly and time-consuming, and often requires extrapolation for extreme events [11,12]. Instead, a low-cost velocity sensor could allow for a permanent installation to collect continuous and real-time velocity data for more accurate discharge information; if truly low cost, then continuous and simultaneous measurements across the stream’s cross section could be obtainable. A wide range of technologies have been explored for new low-cost, low-power velocity sensors, including time of flight measurement, Doppler shift techniques, and velocity dependent force sensing.

Doppler shift techniques have been explored in [13,14]. The authors of [13] demonstrated the feasibility of non-contact measurements of surface velocity using a radar-based sensor and a novel sensor data processing algorithm to extract this surface velocity. The authors focused on a demonstration of this technology as an accurate and low-cost method for measuring surface velocity rather than the implementation of the principle into a field-ready self-contained sensor. The authors of [14] established the potential of utilising visible light as a carrier wave for Doppler-based velocity measurement. It further develops the signal processing methods and provides experimental verification of using visible light in low-cost velocity sensors. Again, more emphasis is applied to demonstrating the principle as opposed to its implementation in deployable sensors.

Low-cost contact and non-contact sensors have been proposed by [15]. Both the contact- and non-contact-type sensors are tested and produce accurate measurements of the water velocity. The contact-type sensor uses a hall effect device to measure the rotational speed of a pinwheel located in the water. The non-contact-type sensor uses video from a digital camera to detect the movement of surface features in particle image velocimetry. These methods, while accurate, are ultimately unsuitable for long-term monitoring applications. For the contact sensor, the moving pinwheel will become clogged and require maintenance, while particle image velocimetry techniques require large amounts of computational resources, leading to high power usage and bulky installation.

Methods based on the velocity dependent force exerted by a water flow on a mast or cantilever are used in [16,17]. The authors of [16] demonstrated that low-cost bidirectional sensing of water flows in a pipe can be achieved by measuring the deflection angle of a thin metallic cantilever in the flow and found that the technique can achieve small error. It is acknowledged that further work on this sensor would be needed for a low-cost and onboard method of measuring this deflection angle. A sensor for a near-bed velocity measurement incorporating the measurement of the frequency of vortex-induced vibrations of a mast submerged in the flow for the determination of velocity is presented in [17]. This novel method was successfully implemented in a low-cost sensor for continuous in-field measurement; however it specialises in measuring the velocity in the immediate vicinity of the channel bed and not the bulk water velocity.

Other velocity methods have been proposed by [18], based on aerial particle image velocimetry, and by [19], based on ultrasonic time-of-flight methods. These require either open-channel and visible flows for the expensive but novel method in [18] utilising a remotely piloted aircraft or full-pipe flows, as in [19]. While these are novel methods to measure velocity, they are not applicable to the smaller scale, underground storm and sanitary sewer systems.

As such, this paper will fill the research gap identified in the literature by developing and validating a low-cost, low-power velocity sensor, which can be used to continuously measure flows in storm and sanitary sewer systems. The sensor’s design is freely available and is based on continuous wave (CW) ultrasonic Doppler; this technology was chosen for its relative simplicity and, thus, abilities to be implemented at low cost and to be optimised for low power. While work discussing velocity sensing have mainly focused on novel measurement principles and their implementation, this sensor is a waterproofed and integrated design that addresses the need for a ‘field-ready’ velocimeter suitable for real-time monitoring of open channel flows able to be deployed at scale. The deployment of the sensor at this scale is supported by its low-cost and low-power usage, which enables it to be installed for long periods of time without maintenance. This research has three major objectives: (1) to develop the low-cost sensor architecture including the circuitry and the sensor case, (2) to develop the sensor data processing algorithm, and (3) to validate the performance of the sensor under typical installation scenarios. The sensors design files and firmware are made freely available under an open hardware licence to allow the reader to construct and modify the sensor design.

## 2. Materials and Methods

**Figure 1:**
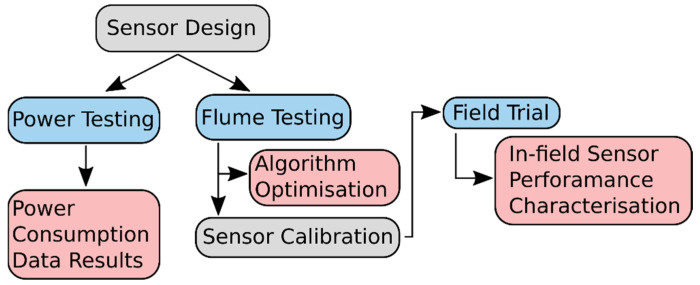
Flowchart describing the design, validation, and characterisation procedures used in this study; major experiments are in blue, and outcomes are in red.

A detailed description of the sensor’s design and design choices is provided. Following this, power testing is conducted to measure the current consumption of the sensor in various operational modes and to verify its low power status. Flume testing of the prototype designs is used to optimise and help develop the digital signal processing algorithm used to extract the velocity measurements. Following sensor calibration, the sensor is deployed in an 8-week field trial to validate and characterise the performance of the sensor in a realistic installation scenario. A flowchart of the studies framework is provided in Figure 1.

### 2.1. Sensor Design

The sensor’s reading is based on CW ultrasonic Doppler. This reflects an acoustic pressure wave off particles and impurities in the water [20]. Based on their velocity, the returning acoustic wave has its frequency shifted by what is known as the Doppler shift ∆f. The equation which governs this phenomenon is

(1) $$\Delta f=\frac{2f}{c}v\cos\theta ,$$

where *f* is the transmitted ultrasonic frequency, *c* is the speed of sound in water, *v* is the flow velocity, and *θ* is the angle between the ultrasonic beam and the water velocity [21]. For the sensor, *f*, *c*, and *θ* are controlled or known from theory. Hence, a measurement of ∆*f* can be used to determine the flow velocity via

(2) $$v=\frac{c\Delta f}{2f\cos\theta }.$$

The relative simplicity of the CW ultrasonic Doppler principle reduces the complexity of the sensor design. This aids in achieving low-cost designs as it both reduces the component count of the design and allows the sensor to be built from non-specialised components, which often do not carry the price premium of more specialised components. To supplement the minimal design of the sensor, digital signal processing (DSP) is used in the measurement of the Doppler frequency shift, ∆*f*. Fourier methods have been shown to be a powerful technique in Doppler frequency estimation, particularly when there is a high signal to noise ratio [22]. Hence, the Fast Fourier Transform (FFT) is utilised to analyse the data in the frequency domain.

**Figure 2:**
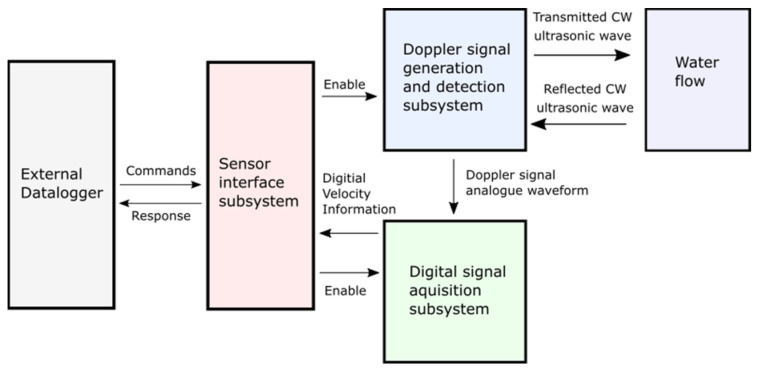
Functional block diagram of major sensor subsystems. Interaction between subsystems displayed on arrows. The sensor consists of the Doppler signal generation and detection subsystem (see Section 2.1.1), digital signal acquisition subsystem (see Section 2.1.2), and sensor interface subsystem (see Section 2.1.3). The sensor interacts with the water via the ultrasonic transducers and is connected to the external datalogger via electrical cable.

**Figure 3:**
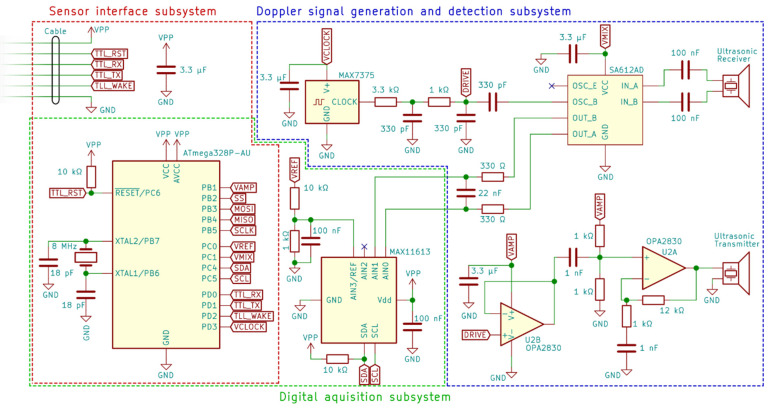
Circuit diagram of sensor. Components corresponding to each subsystem are contained within the dashed line coloured: blue, Doppler signal generation and detection subsystem (Section 2.1.1); green, digital signal acquisition subsystem (Section 2.1.2); and red, sensor interface subsystem (Section 2.1.3). Note that the digital acquisition subsystem and sensor interface subsystem contain components in common. For a complete set of design files, please see the Supplementary Materials.

The sensor has three major subsystems: (1) the Doppler signal generation and detection subsystem, (2) the digital signal acquisition subsystem, and (3) the sensor interface subsystem. Each of these subsystems are described in detailed below. A block diagram of their relationships is provided in Figure 2, and a circuit diagram is provided in Figure 3.

#### 2.1.1. Doppler Signal Generation and Detection Subsystem

**Figure 4:**
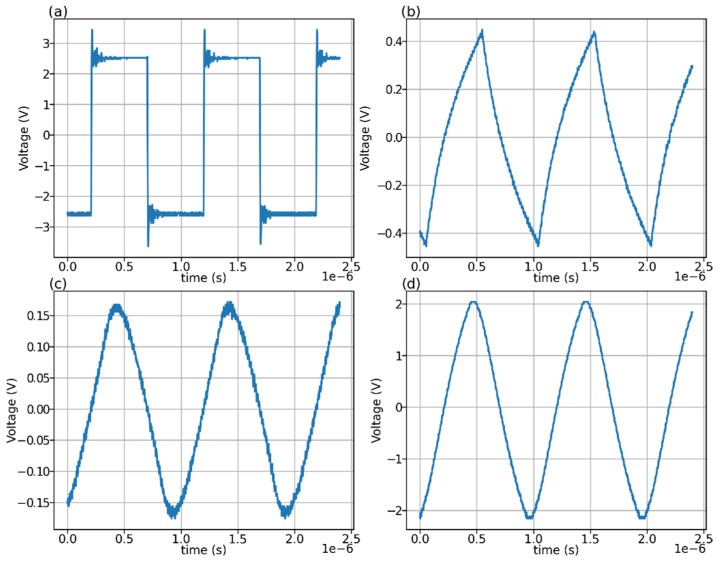
Measured waveforms for Doppler signal generation subsystem. All waveforms recorded with AC coupling at 1 GSa/s. (**a**) MAX7375 output. (**b**) First low-pass filter output. (**c**) Second low-pass filter output. (**d**) Ultrasonic emitter drive signal.

The implementation of the Doppler velocity principle of measurement used in this sensor can best be understood through a procedural explanation. CW ultrasonic Doppler requires a known frequency to be transmitted into the water for the Doppler shift to be measured. The MAX7375 oscillator IC was used as it outputs a 1 MHz 5 V peak-to-peak square wave [23]. A waveform of this output is measured in Figure 4a. The 1 MHz frequency matches the resonance frequency of the SMFM21F1000 ultrasonic transducers used for the emitter and receivers. Each transducer is 12.95 mm in diameter. The signal from the oscillator is filtered through two passive low-pass filters with cut-off frequencies of 150 kHz and 480 kHz. Two filters are used to achieve second order roll-off, supressing the harmonics of the square wave to produce a better approximation of a 1 MHz sine wave. The waveform after the first filter is shown in Figure 4b, and that after the second filter is shown in Figure 4c. The low-pass filters result in a waveform with 6% total harmonic distortion. The choice of this square wave generator over a pure sine wave generator is due to its simplicity, requiring only the low-cost MAX7375 and passive components. The cut-off frequencies of 150 kHz and 480 kHz are below the 1 MHz fundamental frequency and, thus, attenuate the 1 MHz component. This is intentional as it brings the amplitude to an appropriate level for later use in the SA612AD mixer IC.

This signal is then split into an AC-coupled high-bandwidth operational amplifier and into the local oscillator (LO) input of the SA612AD mixer (OSC B). The high-bandwidth operation amplifier is configured as an AC-coupled amplifier that amplifies the high impedance 1 MHz sine wave output of the oscillator to a low impedance 4 V peak-to-peak waveform with 5% total harmonic distortion (Figure 4d). This is then used to drive the ultrasonic transmitter. Other commercial ultrasonic Doppler systems operate on greater voltages of 12 V or higher [24,25,26]. This aids greatly in increasing the output power of the ultrasonic transmitter as it is proportional to the square of the input voltage. The design goals of this sensor are low power and wide compatibility with 5 V microcontroller systems; however, the sensor is restricted to the use of a 4 V signal to drive the ultrasonic transmitter.

The reflected Doppler shifted signal is then detected by the ultrasonic receiver, which outputs a voltage proportional to the incoming acoustic signal. The receiver is connected to the mixer IC via a balanced connection. Internally, the SA612AD uses a Gilbert cell to mix the LO input signal with the received Doppler signal. It also provides an amplification of 6 dB [27]. Theory states that the mixing of two sine waves of frequencies *f*_1_ and *f*_2_ produces a signal with output frequencies of |*f_1_* + *f_2_*| and |*f*_1_ − *f*_2_|. Critically, since the ultrasonic transmitter was driven from the same 1 MHz frequency source, *f*, as the LO input, the input frequencies into the mixer are *f* + ∆*f* and *f*, where ∆*f* is the Doppler shift. Therefore, the mixer outputs at frequencies |∆*f*| and |2*f*–∆*f*|. It is noted that the presence of the | · | in the demodulation removes any information about the direction of flow. This is a limitation compared to other demodulation techniques such as quadrature demodulation; however, quadrature demodulation requires duplicates of many components to accommodate an additional channel [13,28]. This increases the complexity and cost of the sensor. Furthermore, the surface water systems for which the sensor is targeted have unidirectional flows, as such the direction of the water flow does not need to be determined.

The mixed signals are output from the mixer to a balanced low pass filter, which has a cut-off frequency of 11 kHz. The cut-off frequency is chosen to be similar to that of the sampling rate and above the Doppler shift frequencies corresponding to the highest velocities that the sensor can measure. The low-pass filtering is necessary to filter out the signal component of frequency |2*f* − ∆*f*|, preventing aliasing when the signal is digitally sampled. This signal is then output to the digital signal acquisition subsystem.

#### 2.1.2. Digital Signal Acquisition Subsystem

The digital signal acquisition subsystem consists of a differential analogue-to-digital converter (ADC) and the ATmega328P microcontroller unit (MCU). The differential ADC samples the balanced signal output from the Doppler signal generation and detection subsystem. Using a differential ADC over a single sided output enables a 3 dB gain in signal amplitude, critical for aiding in the detection of weak signals. It also eliminates the need for biasing circuitry as the input is not sampled in reference to circuit ground. The ADC samples the signal at a frequency of 11 kHz, along with the DSP, this caps the maximum measurable velocity of the sensor to 1200 mm/s. The ADC converts the analogue signal into a 12-bit signed digital readings. The conversions are sent over an I2C bus to the MCU, where they are recorded for digital signal processing. The digital signal processing algorithm is discussed in detail in Section 2.1.6.

#### 2.1.3. Sensor Interface Subsystem

An ATmega328P microcontroller is used to control subsystems of the velocity sensor, to compute necessary digital signal processing, and to provide a digital I/O interface. The use of the ATmega328P was selected as it is a low-cost solution to managing these tasks; is relatively simple to program for; and is compatible with the Arduino IDE, a popular and easily accessible platform for programming MCUs. The latter two reasons aid in making the design more accessible to end users who want to modify the sensor to better fit their application but are relatively unfamiliar with MCU programming. For lower power consumption and higher signal processing performance, the ATmega328P may be substituted for an STM32L412 or similar.

To reduce power consumption the sensor is kept in a low power sleep mode with the Doppler signal generation and detection subsystem and parts of the digital signal acquisition subsystem isolated from the supply voltage. When the microcontroller receives a request for a velocity measurement over the IO interface, these systems are woken up to begin a measurement. This helps significantly with reducing the current consumption of the sensor, particularly when measurements are temporally sparse.

The sensor communicates to a datalogger via a USART interface at a baud rate of 9600. This has been proven to be stable for cables up to 20 m in length. Commands can be sent to the sensor, including to make a velocity measurement, query the sensor ID and firmware version, or send the sensor to sleep. There is also a wake line necessary to be pulsed to wake the sensor from its low power sleep state. A reset line is also available. This allows the sensor to be reprogramed, to add more functionality, or to customise the sensor to the end users’ requirements. Power is provided through a 5 V and GND line to the sensor.

#### 2.1.4. Physical Overview

**Figure 5:**
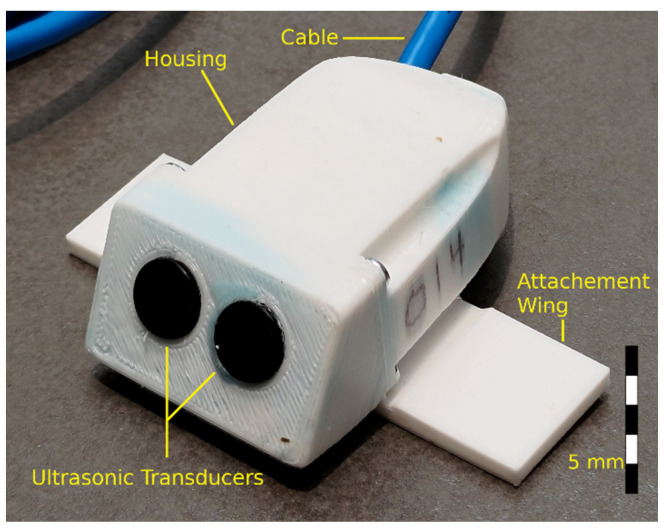
Picture of the velocity sensor with approximate scale overlayed. Key external features of the sensor have been labelled. The sensor measures 75 mm by 44 mm by 25 mm without the optional attachment wings.

The sensor’s housing was constructed using 3D printing technology. It is printed out of a PET-G filament. An image of the sensor is provided in Figure 5. The housing is filled with a two-part potting mixture, which waterproofs the sensor’s electronics. The front of the sensor contains the two ultrasonic transducers, which are angled such that their acoustic beam is elevated 30 degrees from the horizontal. This is done to have the reading obtain information from a larger range of flow depths as opposed to if the transducers were angled in the horizontal. It has been shown that this orientation gives a more representative reading of the mean flow velocity and with less dependence on the water depth [29]. The optional attachment wings aid in affixing the device to a channel bed via an anchor bolt. The wiring for the sensor exits through a CAT5 four-pair cable. The total current cost to manufacture the sensor is less than 50 USD. As the sensor is only currently in boutique production, it is thought that this could be further reduced with mass production techniques.

#### 2.1.5. Calibration

Calibration of the sensor is necessary to quantify its background noise profile, that is the measurement at no flow. This profile is then filtered out of the sensor data as part of the DSP. Specifically, a power spectral density (PSD) that quantifies the amount of noise at each frequency in the digitised Doppler shift signal is estimated. The variability in the construction of the sensor is such that each sensor requires its own calibration before deployment. To measure this PSD, the sensor is placed in still water. The transducers are oriented such that there is at least 20 cm of unobstructed water in front of them. Then, 256 samples are taken from the ADC at a sampling frequency of 11 kHz. At least 2000 of such 256 sample segments are taken. Welch’s method is then used on these segments with a 0% overlap and hamming windowing to produce the PSD estimation [30].

#### 2.1.6. Digital Signal Processing Algorithm

**Figure 6:**
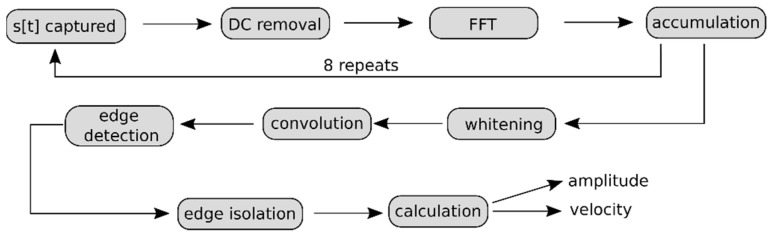
Digital signal processing flowchart. Each step is displayed in the grey boxes. Example data for the processed signal after certain steps can be found in Figure 7. The algorithm begins at *s*[*t*] captured.

**Figure 7:**
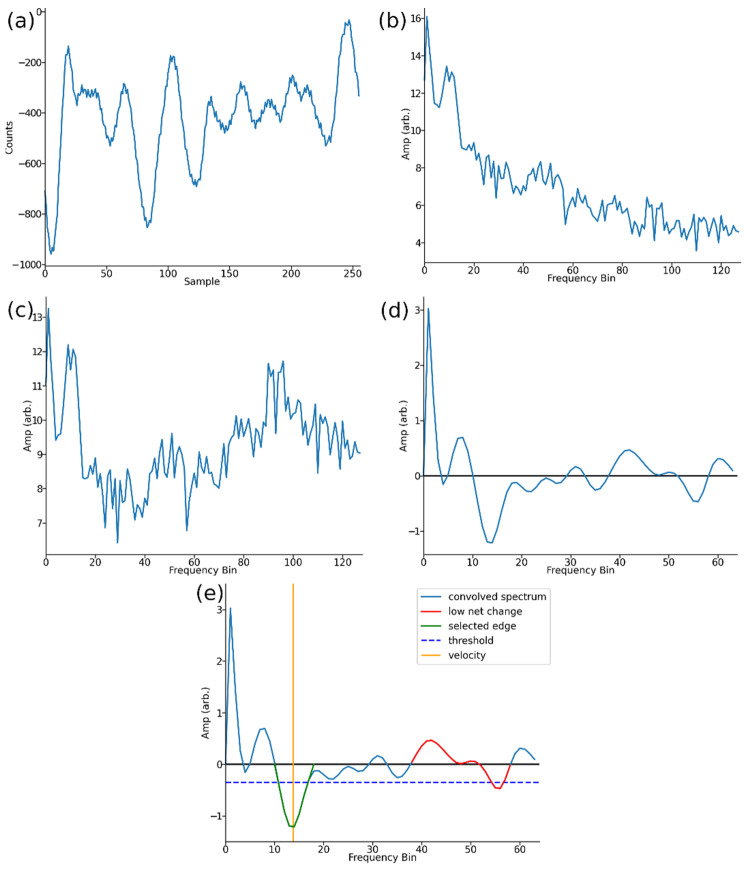
Signal waveform after points in digital signal processing pipeline: (**a**) raw *s*[*t*] captured data, (**b**) waveform after FFT accumulation and logarithm, (**c**) waveform after whitening, (**d**) waveform after convolution, and (**e**) waveform showing edge detection and final velocity reading. The edge detection threshold is shown in dashed blue, an edge rejected due to a low net change is shown in red, and the selected edge is shown in green. The orange vertical line displays the frequency bin corresponding to the final measured velocity.

The principle task of the digital signal processing algorithm is to estimate the central Doppler signal from the sampled data to calculate flow velocity. This is a task made more challenging by the high SNR environment. The algorithm is run on the MCU and is thus subject to considerable memory and processing power constraints. Many Doppler velocity sensors determine the Doppler frequency by calculating the mean of the measurement power spectrum. For this sensor, it was found based on laboratory flume testing (Section 2.2.2) that an algorithm that looks for the dominant falling edge in the reflected power spectrum performs better at obtaining the Doppler frequency. A flowchart overview of this algorithm is provided in Figure 6. Let *s*[*t*] be *t*-th sample in the array of data captured by the ADC from the received signal (Figure 6, *s*[*t*] captured, and Figure 7a). An algorithm to remove the DC component of s[*t*] is applied. Under this algorithm the array is updated,

(3) $$s\left[t\right]\;:=\;s\left[t\right]-\langle s\rangle ,$$

where ⟨*s*⟩ is the arithmetic mean of all samples, *s*[*t*] (Figure 6, DC removal). The data are then transformed via an FFT to convert to convert it to the frequency domain. This yields an array of frequency domain data, *S*[*f*]. As *s*[*t*] was purely real, then *S*[*f*] is symmetric about its midpoint, the 128-th element. The data beyond the 128-th element in *S*[*f*] are therefore discarded to save memory (Figure 6, FFT).

The square of the magnitude of each element in *S*[*f*] is then accumulated into a new array *S_acc_*[*f*], according to the following:

(4) $$S_{acc}\left[f\right]+=S\left[f\right]S^{*}\left[f\right],$$

where ∗ denotes the complex conjugate and *S_acc_*[*f*] is initialised to zero for the first dataset. New data are sampled and accumulated into *S_acc_*[*f*] for a total of eight times (Figure 6, accumulation). This is done to calculate the average power spectrum density (PSD) over a short period of time, reducing the variability seen in any single measurement. Once this is complete the square root of each element is taken,

(5) $$S_{acc}\left[f\right]:=\sqrt{S_{acc}\left[f\right]}$$

To give a final PSD, for debugging and testing purposes, the PSD at this point is sent to the datalogger and recorded on an SD card. The next steps of the algorithm are dedicated to estimating the Doppler frequency of the recorded spectrum in *S_acc_*. The algorithm attempts to detect the frequency at which a falling edge in the PSD occurs, that is where the reflected signal due to the particles in the water significantly drops off in power.

The natural logarithm of each element *S_acc_*[*f*] is taken (Figure 7b), and then, the array is whitened according to

(6) $$S_{acc}\left[f\right]:=S_{acc}\left[f\right]-C\left[f\right]$$

where *C*[*f*] is the sensor’s calibration PSD and corresponds to the relative background noise at the frequency *f* (Figure 6, whitening and Figure 7c). *C*[*f*] is determined as in Section 2.1.5.

*S_acc_* is then convolved with a Gaussian derivative kernel of standard deviation 2 bins to produce array, *D_acc_*. The edge data in *S_acc_* are now present as peaks in the *D_acc_*, and the use of the Gaussian derivative helps to inhibit the effect of noise appearing as an edge by smoothing the data. The values *D_acc_* [*f*] for *f* = 0, 1, 2 and *f* > 64 are discarded. This is due to a high SNR for these frequency bins due to the inherent noise of the sensor (Figure 6, convolution and Figure 7d).

The last local minimum (highest frequency) of *D_acc_* above a pre-set threshold is found; this corresponds to a falling edge. Let this occur at *D_acc_* [*f′*]. A check is then conducted to test if this edge corresponds to a sustained increase in the reflected power or a ‘bump’. This is achieved by calculating the net change in *D_acc_* over the found falling edge at *f′* and previous rising edge, if this net change is below a given threshold, the edge is discarded and a prior falling edge at a lower frequency is considered. This is repeated until a falling edge is found, which fulfils the above criteria (Figure 6, edge detection).

Once a falling edge that meets the required condition is found, the falling edge is isolated; that is, only the data of *D_acc_* between the zero crossings on either side of *D_acc_* [*f′*] are considered (Figure 6, edge isolation and Figure 7e). Finally, the height difference in the edge is assigned to the signal strength of the datapoint and the mean frequency of the isolated peak in *D_acc_* is taken to be the Doppler frequency. A conversion between Doppler frequency and velocity is performed via (2) (Figure 6, calculation).

### 2.2. Lab and Field Testing Setup

#### 2.2.1. Lab Power Usage Testing

For measurements of the power usage of the sensor, an ammeter was placed in series with the 5 V supply line; the sensor was then connected to an Arduino Uno [31], from which the sensor received its power and communications. Three different power states were investigated: measurement, active, and sleep, with these representing typical states in the operation of the sensor. The measurement state had all devices in their full power states, as would be the case when a measurement is occurring. The active state had the devices in the digital signal acquisition and sensor subsystem in their active states and devices in the Doppler signal generation and detection subsystem powered down. Hence, in this state, the device is ready to accept commands. In the sleep state, all devices were in their low power states. For each of these states, the current displayed on the ammeter was recorded. Commands over the UART were used to switch between the power states.

#### 2.2.2. Flume Testing

To aid in the development and prototyping of both the sensor and signal processing algorithms, laboratory tests were conducted. In this test series, the velocity sensor was installed facing up-stream near the invert of a flume channel so as to remain submerged through the tests. The flume was able to produce flow velocities ranging from 100 to 400 mm/s and was measured using the Flo-Mate Model 2000 portable flow meter also mounted in-line with the velocity sensor. At various flow rates, diagnostic engineering data were collected from the sensor, including raw transducer output, ADC samples, and formatted velocity data. The results from successive tests were used to inform the design of the sensor hardware and algorithm. The results will not be discussed here as they were conducted with previous iterations of the sensor. To evaluate the sensor’s real-world performance and to overcome the limited flow velocities achievable in the laboratory setup, we focus our performance study on in-field testing.

#### 2.2.3. Field Trial

For testing, the sensor was installed at the bed of Dandenong Creek, Australia, facing upstream and with the ultrasonic detection cone positioned azimuthally. The sensor was set to a measurement interval of 2 min. It was connected to an Arduino Uno datalogger, which recorded the averaged FFT data of the sensor after each measurement. Installed next to the sensor was a HACH Submerged Area Velocity Sensor [24]; this logged the velocity of the water every minute and was used as the source of comparison for the sensor under test. The data points from the sensor and those from the HACH probe were associated by pairing the datapoints nearest in time. For the analysis, it was assumed that HACH probe had negligible uncertainty, which, while we acknowledge is not correct, was carried out to compare our low-cost sensor to this well-established, industry-leading sensor. The weekly retrieval of data from the site was conducted for eight non-consecutive weeks beginning 2020 July and ending in 2020 October. The velocity sensor received its power from the Arduino Uno datalogger over a 20 m cable, which was also used for data transfer between the sensor. The collected data from the sensor was the processed externally using the described algorithm to obtain the velocity and signal strength reading. This was carried out to aid in adjusting the algorithm for Doppler frequency estimation. For the data analysis, a filter was applied to the sensor’s data to remove low-signal-strength points with velocities higher than 250 mm/s. Low-signal-strength high-velocity readings occur mostly at low water depths [32]. As they are not an accurate measure of the water velocity and are clearly distinguishable from the typical high signal strengths seen at high velocities, they are filtered from the analysed data set.

## 3. Results

### 3.1. Lab Power Usage Testing

A sleep current of 100 µA was found for the sensor. This moderately low sleep current supports the design criteria of the sensor being compatible for long-term instalment as a low-maintenance real-time monitoring device (Table 1). Given the measurement time of 4 s, supply voltage of 5 V, and measurement current of 27 mA, this leads to an energy per measurement of just 0.6 J. For a measurement regime with 10 measurements per hour, a calculated total consumption over 1 year of logging is 3500 mAh, about the typical capacity of an 18650 Li-ion cell. A lower measurement frequency would extend the logging lifetime; however, at lower measurement frequencies, the proportion of current used in sleep, which depends only slightly on the logging interval, would increase from the 25% in the above scenario.

At 10 measurements per hour, the average continuous power consumption is 2 mW. This compares very favourably to current state-of-the-art commercial sensors. This information is summarised in Table 2. Both the continuous current consumption and the energy per measurement of the sensor are reduced by more than an order of magnitude compared to the HACH Submerged Area/Velocity Sensor. The continuous power consumption of the sensor is also lower than the 50 mW to 500 mW of the OTT SLD and 20 mW of the SonTek-IQ. This reduction in power usage enables smaller batteries to be used in remote logging applications. This facilitates the lower-cost and easier installation of the sensor when compared to current solutions.

There is also scope for the power consumption of the sensor to be reduced further. While fundamental properties of the FFT algorithm used necessitate a minimum amount of time for which data need to be collected to maintain the velocity resolution of the sensor, giving a lower bound to measurement time, this lower bound has not yet been achieved due to the large portion (3.5 s) of time spent computing the signal processing algorithms to extract the velocity. Future work could involve optimisation of this algorithm to bring further power savings.

### 3.2. Field Trial

**Figure 8:**
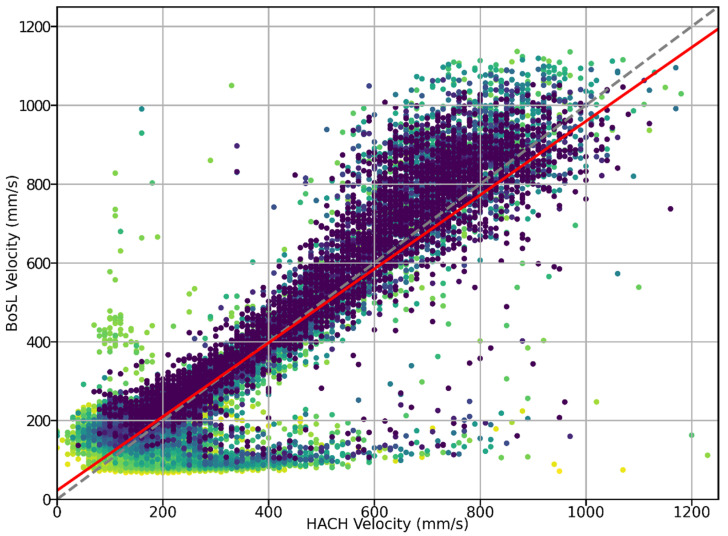
Plot of the sensor measured velocity on the y-axis vs. HACH probe measured velocity on the x-axis. Points are coloured according to signal strength, with darker points having a higher signal strength. Red: line of best fit with gradient 0.94 and y-intercept 22 mm/s, R^2^ value 0.78, probability of null hypothesis of gradient equal to zero *p* < 0.0001. Linear regression calculated using SciPy [33]. Grey dashed line: 1:1 correlation. A few data points are beyond the plot range; the full data extends to 3000 mm/s on the x axis.

A correlation plot between the sensor measured velocities and velocity as measured by the HACH probe is shown in Figure 8. Both probes indicate that most of the velocities ranged from 0 mm/s to 1200 mm/s. The data display a linear trend for flow velocities up to 1200 mm/s—the measurement ceiling of the sensor. Calculating a line of best fit gives a gradient of 0.94, an R^2^ value of 0.78, and the probability of the null hypothesis that the gradient is zero is *p* < 0.0001, supporting the sensor can accurately measure the velocity. Whilst a linear trend is present, there are also other qualities to the velocity measurements. It is seen that there are a cluster of points at a sensor’s velocity from 100 mm/s to 200 mm/s and a very wide range of HACH velocities. That is, the sensor may sometimes significantly underestimate the flow velocity according to the HACH sensor (17% of datapoints) and this underestimation occurs more frequently at low velocities rather than at higher velocities. Significant overestimation of the HACH velocity (sporadically high readings) are seen much less commonly. It is important to note that this is in part due to the filtering of datapoints with low signal strengths, as before this filtering, there was a large clustering of great overestimations at low flow velocities. It is possible that further optimisation of the signal strength filter could achieve a more reliable measurement by eliminating a greater proportion of measurements that significantly underestimate or overestimate the flow.

**Figure 9:**
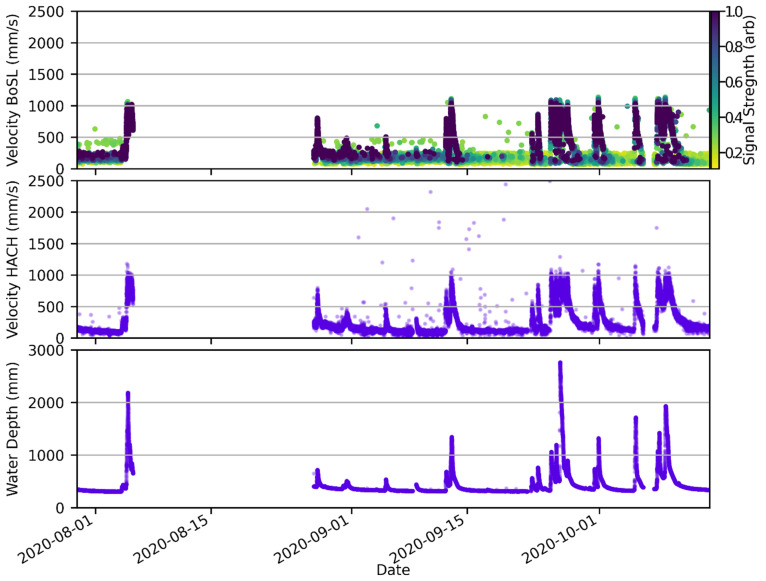
Top: Plot of the velocity measurement of the sensor over time. Middle: HACH probe velocity measurements over time. Bottom: HACH probe depth measurements over time. The x-axis on all plot is the same. There was a pause in data collection from 2020 AUG 5 to 2020 AUG 27. A few data points are beyond the plot range; the full data extends to 3000 mm/s on the y-axis.

To better characterise the behaviour of the sensor, it is also helpful to plot the velocity measurements against time (Figure 9). The sensor records similar velocity measurements to the HACH probe during flow events. Importantly, the maxima of the flow events as recorded by the sensor in this work and the HACH sensor are similar. This indicates that the upper measurement range of the sensor is sufficient to capture the event and not clip the data. It is confirmed here that the sporadically high readings occur almost entirely at low depth, which is a typical feature of ultrasonic Doppler-based velocity sensors [20]. Additionally, the density of sporadically high points is comparable to that of the data from the HACH sensor. Figure 9 also highlights that the minimum velocity at which useful velocity measurements are obtained is at the filter cut-off of 250 mm/s, since below this, velocity readings from the sensor are distributed from 0 to 250 mm/s regardless of the HACH measurement. It is known that the HACH sensor performs multiple velocity scans per recording interval to improve its measurement accuracy, and it is poorly documented whether the resultant velocity output is processed further based on past measurements. If so, this would likely increase the ‘smoothness’ of the HACH data when compared to the velocity sensor.

The root mean square (RMS) error for the sensor’s measurement when compared to the HACH measurement was calculated for measured flow velocities in 200 mm/s bins (Table 3). This was performed to obtain a better description of the measurement error at different flow velocities. The RMS error falls below 30% above flow velocities of 400 mm/s. Larger errors occur at flow velocities below 400 mm/s, particularly below 200 mm/s; however, this is expected as velocities below 250 mm/s are out of the sensor’s designed measurement range. The mean error for the sensor is least at above 400 mm/s, where it remains within ±11%. Below 400 mm/s, particularly 200 mm/s, the mean error increases in magnitude significantly, though still remaining less than 30%.

To further understand the performance of the sensor, causes for the error of the sensor should be analysed. The accuracy of the HACH probe used to compare the sensor’s measurements to is a clear first consideration. The HACH corporation claims a measurement error of less than 3% in ideal conditions [24]. Studies using data from the HACH probe in real-world conditions, however, claimed that the uncertainty could be underestimated significantly, especially at low water velocity, and therefore have taken a more conservative estimate of ± 100 mm/s [34,35]. This absolute uncertainty would contribute significantly to the relative uncertainty estimated at low flow velocities and may partly explain the high error seen at the 0 to 200 mm/s and 200 to 400 mm/s velocity ranges (representing an RMS error of 50% for 200 mm/s and 25% for 400 mm/s). At higher velocities this error from the HACH probe will contribute less significantly to the uncertainty in the measured error for the velocity sensor.

It should also be reiterated that it is not well-documented what post processing the HACH sensor performs on its data. It is a common practice to perform data cleaning of the velocity measurements both to overcome the random error from the sensor and to reduce high-frequency variations in the water velocity induced by pulsation and turbulence [36]. Use of the data should perform some similar data processing, which will result in a reduction in the RMS error.

The sensor does not compensate for temperature. It is hypothesised that temperature could affect the velocity measurement through changes to the speed of sound in water or by altering the frequency of the LO. At 20 °C, the speed of sound in water is 1480 m/s with a variation of 3 m/s·K [37]. The speed of sounds affects the velocity measurement; however, over an extreme 20 °C swing in temperature, a 5% error of the velocity measurement occurs. Likewise, the MAX7573 oscillator used has temperature dependence on its output frequency; this similarly affects the velocity. However, a 20 °C swing here produces a less than 1% variation in output frequency [23]. The sensor also compares favourably to other current works on velocity sensing. The low-cost contact- and non-contact-type water velocity sensors presented in [15] have root sum of squares errors of 10% and 12% over six data points over a range of 0.21 to 1.4 m/s for the contact type and 0.2 to 0.5 m/s for the non-contact type. While these errors are smaller than those for the sensor presented here, they are not too dissimilar and the small number of data points limits the confidence of any conclusion. Furthermore, the sensor presented here has the advantage of containing no moving components, which are likely to be blocked by debris, limiting the reliability of the sensor, or the computationally expensive and high power algorithms needed for particle image velocimetry methods.

Another non-contact method for velocity sensing via a flying optical-based method can be found in [18]. It finds an uncertainty from 5% to 26%. While this sensor performs better at lower velocities, the overall uncertainty range is similar to the sensor in work. The design produced by [18] also serves a different use case of instantaneous measurement of a large number of sites rather than the long-term real-time monitoring application that is the focus of this work.

The recent low-cost hydro-mast for measurement of the velocity near the bed of the channel [17] was tested with a similar field validation method by comparison against a commercial acoustic Doppler sensor. In the velocity range from 0.35 to 1.2 m/s, the sensor was found to have a mean error of at most 95 mm/s. This is just slightly lower than the maximum mean error found for this sensor at 130 mm/s (−11% at 1200 mm/s). Again, while the sensor from [17] is valuable for understanding the flow dynamics in the near-bed. The sensor proposed here measures the mean velocity, more suitable for obtaining a reading representative of the flow across the channel. As discussed, our sensor compares favourably in performance to recent developments in the water sensing space and fulfills the gap of a sensor suitable for long-term, low-cost, real-time monitoring.

Future work should focus on studying the sensor in a large-scale deployments for real-time monitoring and should investigate the benefits and considerations of the sensor to this application. Incremental improvement to the sensor’s design should also be another focus of future research. This may include enhancement of the sensors’ electronics and data processing to increase the sensors’ accuracy, performance at lower velocities, and data reliability.

## 4. Conclusions

The sensor presented has been shown to effectively measure the water velocity via data from an in-field test. The performance of the sensor is best at 400 to 1200 mm/s, where RMS error is below 29% and mean error ranges from −11% to +10%. While this does not match the manufacturer’s quoted performance of current commercial sensors in ideal lab conditions, the performance becomes significantly more comparable in field applications, where there is an increase to the uncertainty. The novelty of the sensor is in enabling the measurement of velocity at a cost of 50 USD, significantly cheaper than the typical thousands of dollars for commercial sensors. Along with the low power usage of the sensor—2 mW for 10 measurements an hour—and its compact size, it supports the deployment of the sensor for real-time monitoring in large quantities. This opens up the possibility to new scientifically interesting monitoring schemes, such as high spatial and temporal data resolution collection in environmental monitoring programs.

## Figures and Tables

**Table 1:** Results of lab power usage testing. Measured current consumption for velocity sensor in various power modes.

Mode	Current
Measurement	27 mA
Active	12.8 mA
Sleep	100 µA
Yearly power use (6 min measurement intervals)	3.5 Ah

**Table 2:** Comparison of power usage of velocity sensors to state-of-the-art commercial sensors. Data for commercial sensors are taken from manufacturers documentation. Continuous power consumption is the average power used during use. For the sensor in this work, this is 10 measurements per hour. The range of continuous power consumptions for the OTT SLD is due to variable measurement frequency.

Power Usage	This Work	HACH Submerged Area/Velocity Sensor [24]	SonTek-IQ [25]	OTT SLD [26]
Energy per measurement	0.6 J	<15 J	not specified	not specified
Average continuous power consumption	2 mW	<1.2 W	>20 mW	50–500 mW
Supply voltage	5 V	9–15 V	9–15 V	12–16 V

**Table 3:** Sensor error characterisation. RMS error calculations for the sensor measurement in in-field testing. A negative mean error corresponds to the sensor under-reading. Data are binned according to the velocity recorded by the HACH sensor.

Velocity Range (mm/s)	Number of Points	RMS Error (%)	Mean Error (%)
0 to 200	15,867	65	+30
200 to 400	6204	43	−23
400 to 600	2463	29	−3
600 to 800	2516	23	+10
800 to 1000	1130	17	+1
1000 to 1200	70	20	−11

## Data Availability

The data presented in this study are openly available in the Supplementary Materials.

## Supplementary Materials

The following supporting information can be downloaded at: https://www.mdpi.com/article/10.3390/s22197451/s1. Please find complete design files necessary to modify and build the sensors along with the data used in the analysis of this study in the Supplementary Materials.
