# Supplementary figures and images for: A Low-Cost, Low-Power Water Velocity Sensor Utilizing Acoustic Doppler Measurement
Source: Sensors (Basel). 2022 Sep 30;22(19):7451. doi: 10.3390/s22197451 (PMC9572237; doi:10.3390/s22197451)

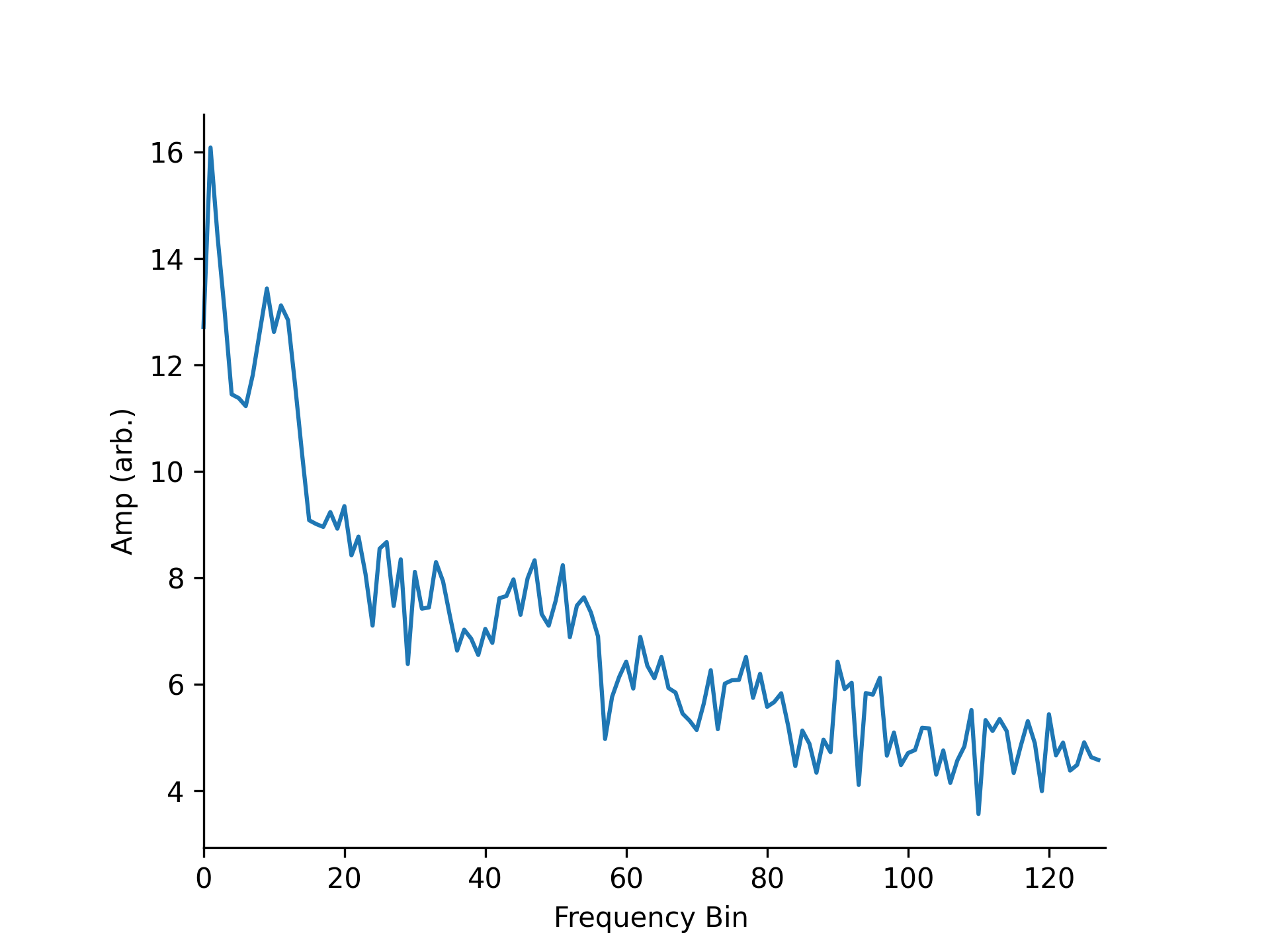

Supplement: Supplementary file 1 [file sensors-22-07451-s001.zip › supplementary_material/data/field_test/fg/acced.png]

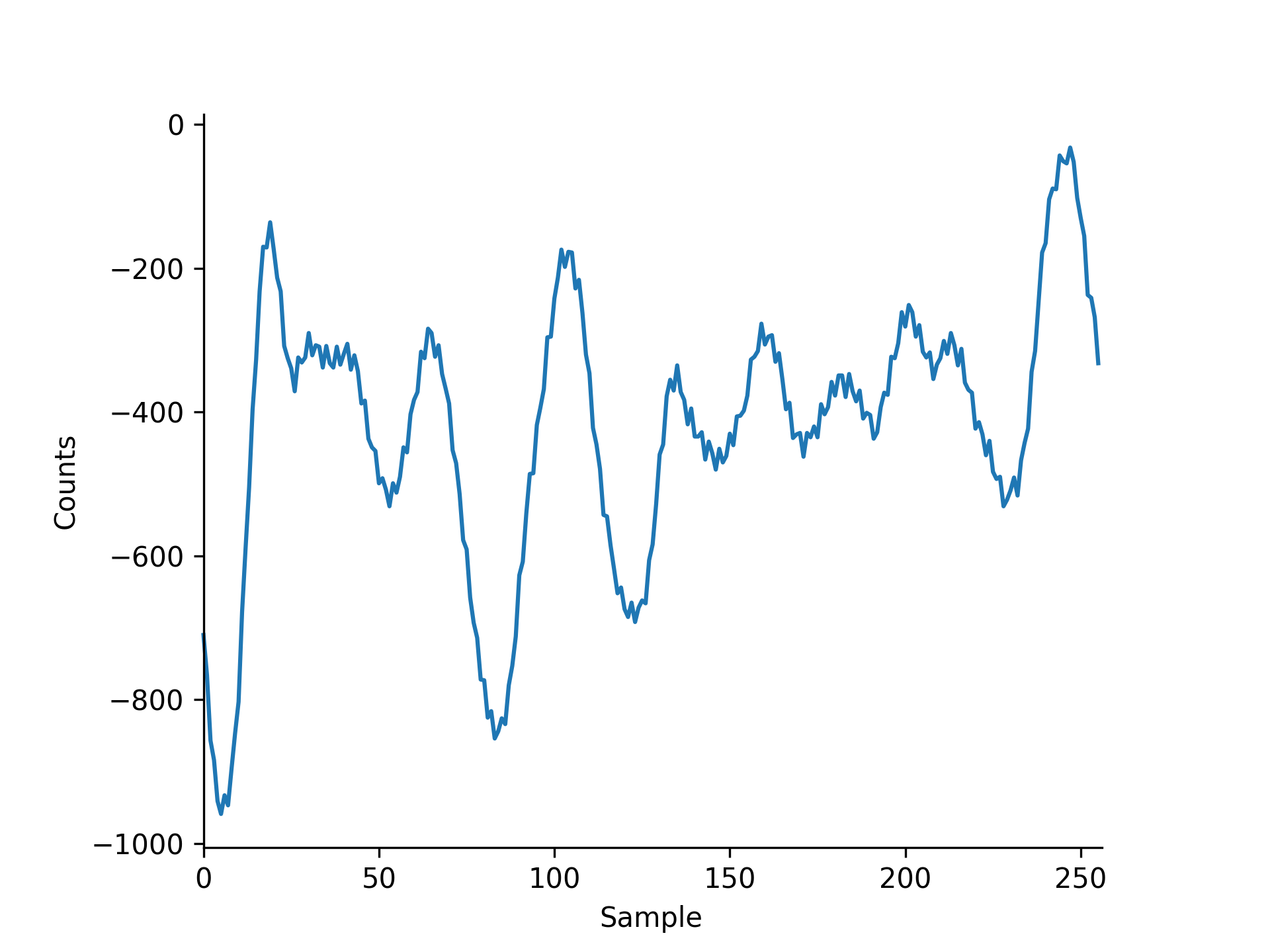

Supplement: Supplementary file 1 [file sensors-22-07451-s001.zip › supplementary_material/data/field_test/fg/capture.png]

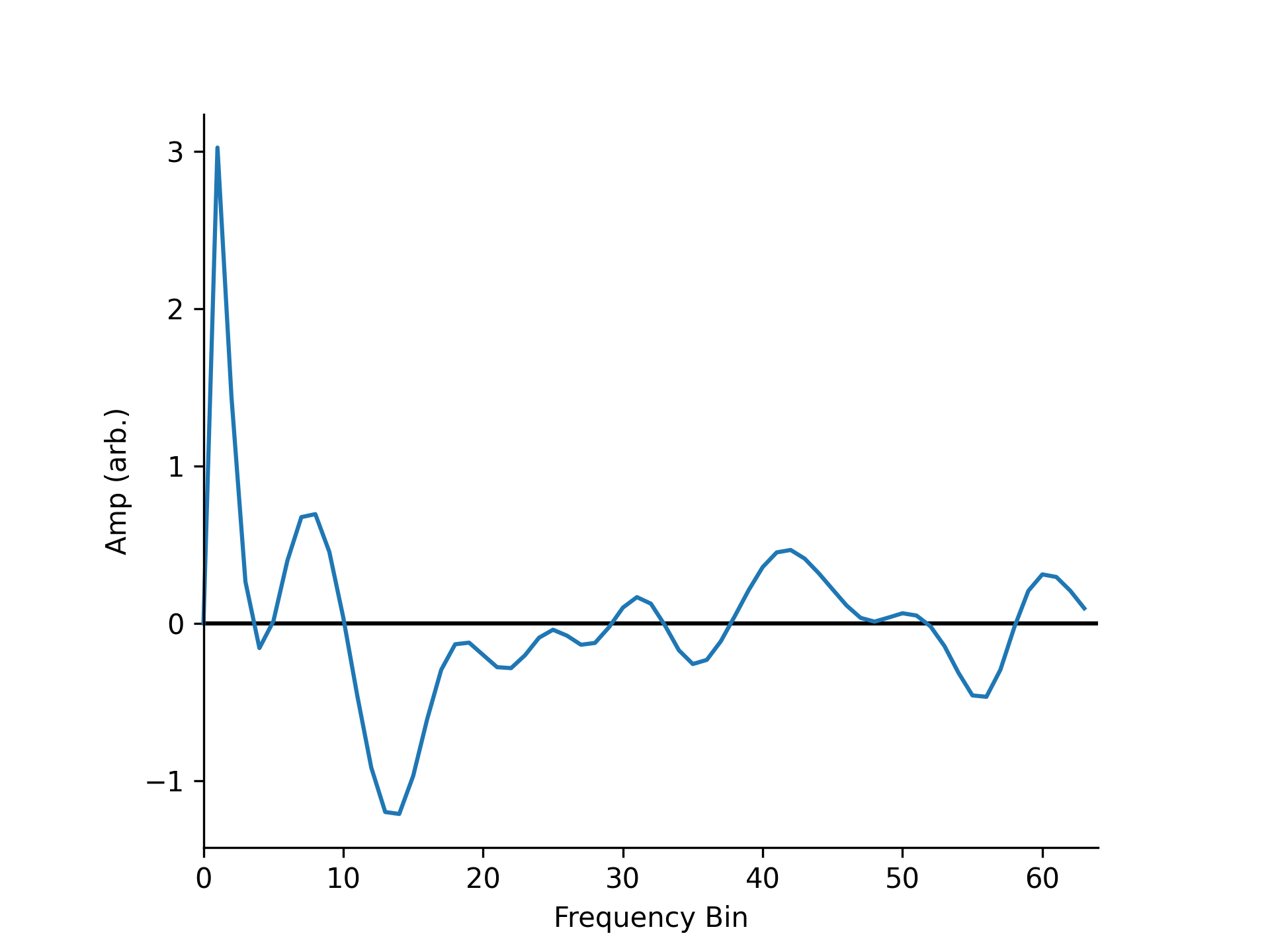

Supplement: Supplementary file 1 [file sensors-22-07451-s001.zip › supplementary_material/data/field_test/fg/conv.png]

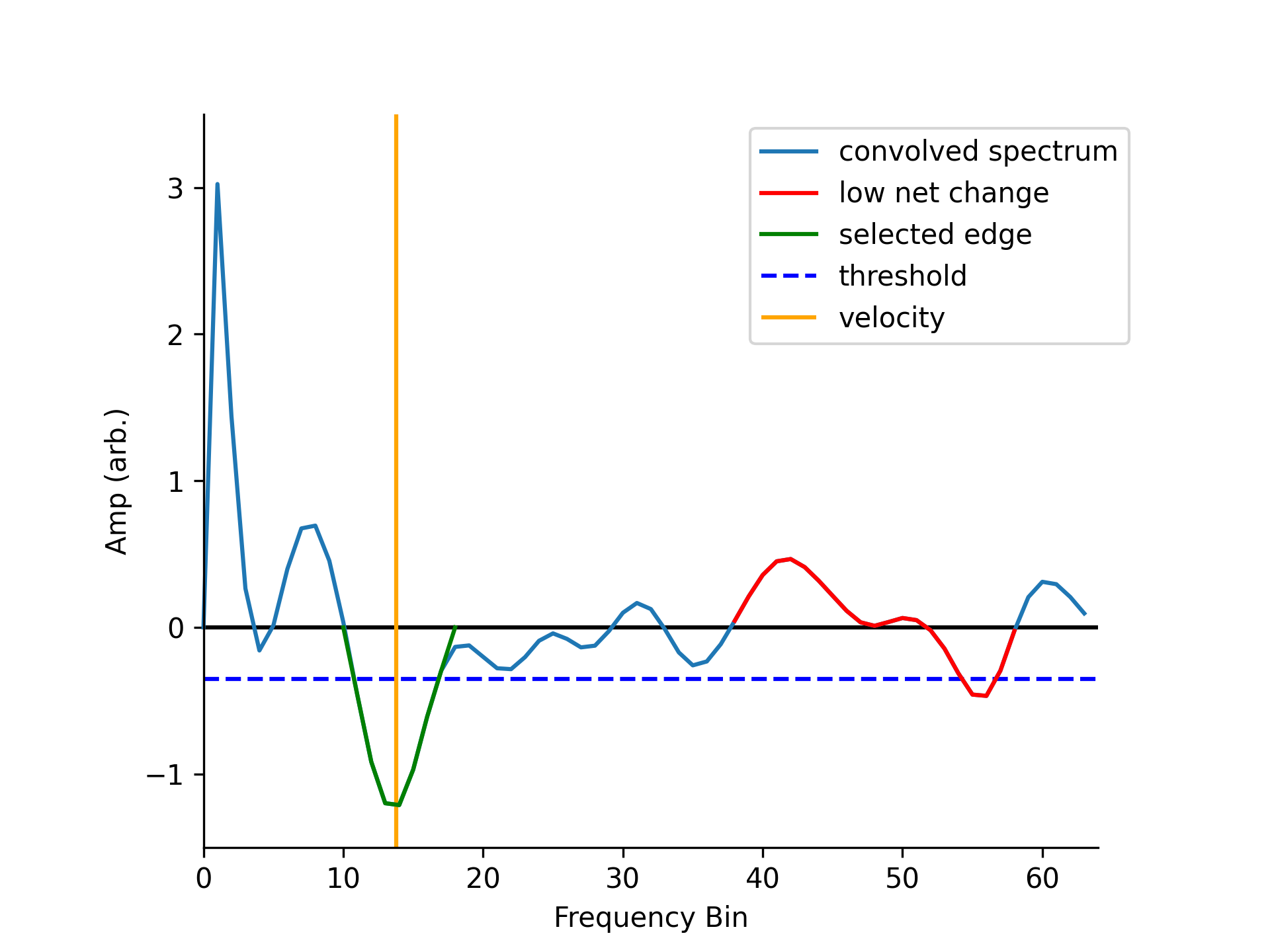

Supplement: Supplementary file 1 [file sensors-22-07451-s001.zip › supplementary_material/data/field_test/fg/edge.png]

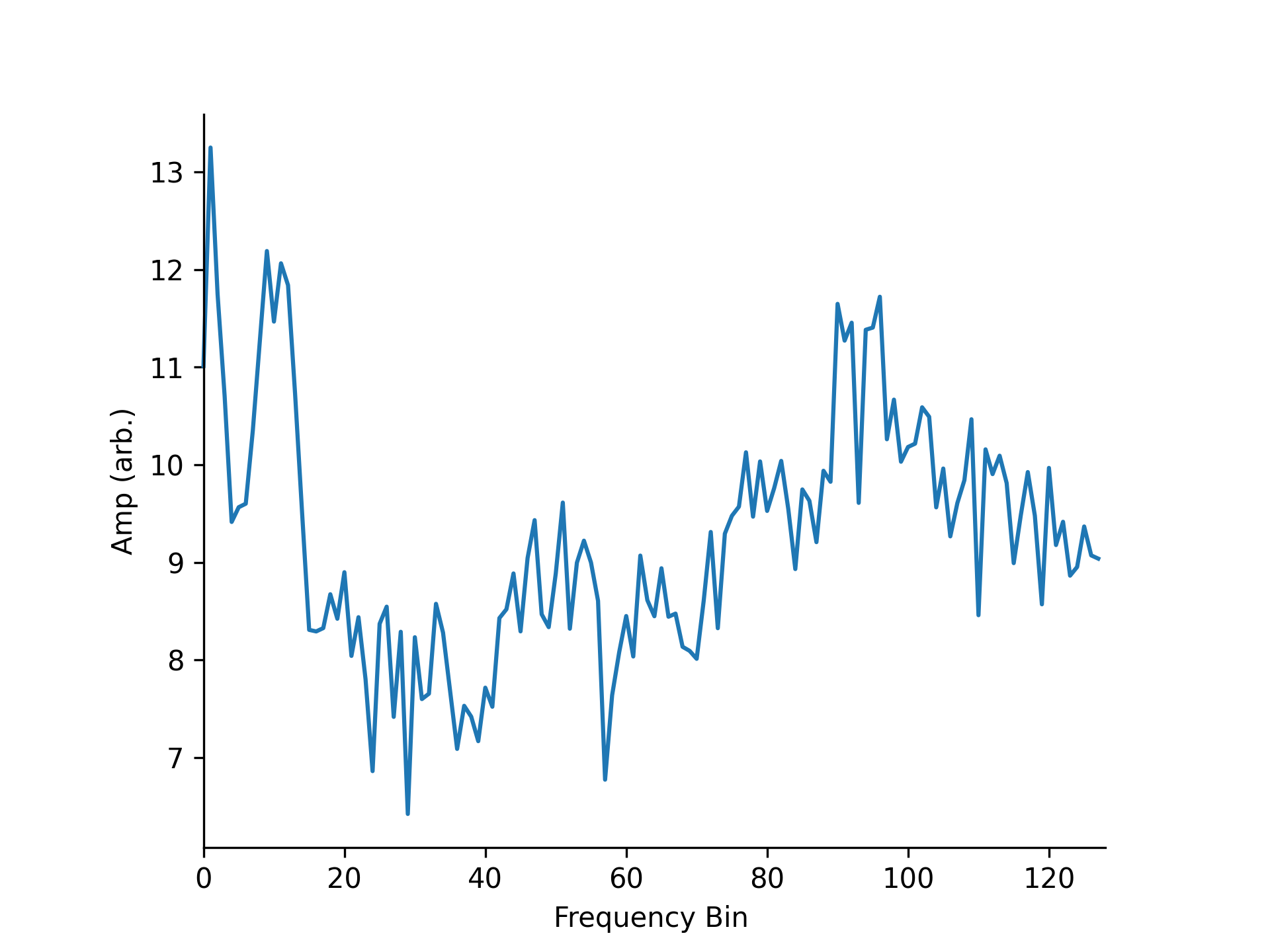

Supplement: Supplementary file 1 [file sensors-22-07451-s001.zip › supplementary_material/data/field_test/fg/white.png]

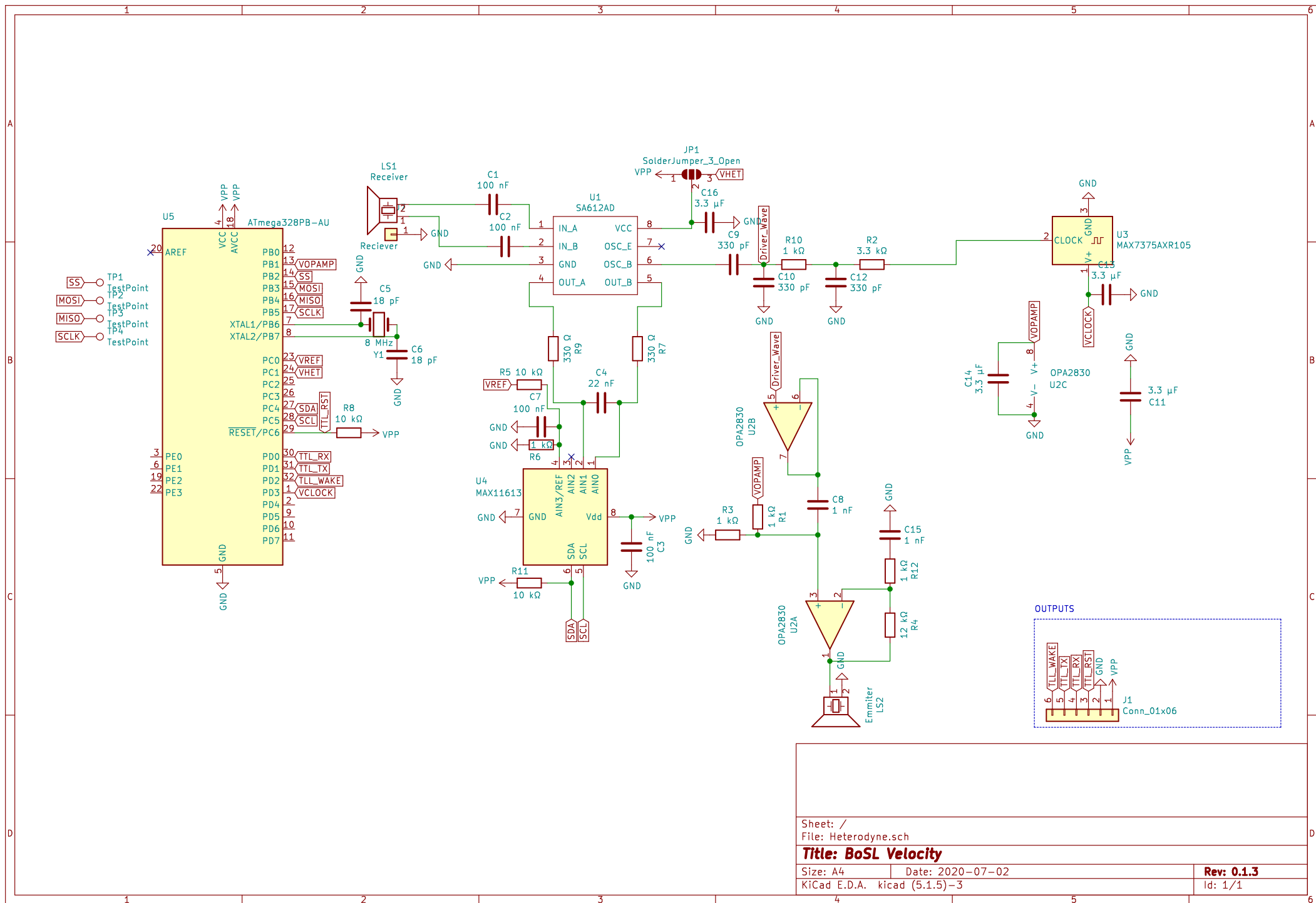

Supplement: Supplementary file 1 [file sensors-22-07451-s001.zip › supplementary_material/EDA/schematic_rev_0.1.3.pdf]
